# Changes of salivary biomarkers under different storage conditions: effects of temperature and length of storage

**DOI:** 10.11613/BM.2019.010706

**Published:** 2019-02-15

**Authors:** Tomás Barranco, Camila P Rubio, Asta Tvarijonaviciute, Mónica Rubio, Elena Damia, Elsa Lamy, Ramón Cugat, José J Cerón, Fernando Tecles, Damián Escribano

**Affiliations:** 1 Interdisciplinary Laboratory of Clinical Analysis (Interlab-UMU), Campus of Excellence Mare Nostrum, University of Murcia, Murcia, Spain; 2 Department of Animal Medicine and Surgery, University CEU Cardenal Herrera, Valencia, Spain; 3 Institute of Mediterranean Agricultural and Environmental Sciences (ICAAM), University of Évora, Évora, Portugal; 4 Arthroscopy and Traumatology Unit of the Quirón Hospital, Barcelona, Spain; 5 Department of Animal and Food Science, Faculty of Veterinary Medicine, Autonomous University of Barcelona, Barcelona, Spain

**Keywords:** biomarker, enzyme stability, oxidative stress, saliva, temperature

## Abstract

**Introduction:**

In this report, we aimed to examine the stability of various analytes in saliva under different storage conditions.

**Materials and methods:**

Alpha-amylase (AMY), cholinesterase (CHE), lipase (Lip), total esterase (TEA), creatine kinase (CK), aspartate aminotransferase (AST), lactate dehydrogenase (LD), lactate (Lact), adenosine deaminase (ADA), Trolox equivalent antioxidant capacity (TEAC), ferric reducing ability (FRAS), cupric reducing antioxidant capacity (CUPRAC), uric acid (UA), catalase (CAT), advanced oxidation protein products (AOPP) and hydrogen peroxide (H_2_O_2_) were colorimetrically measured in saliva obtained by passive drool from 12 healthy voluntary donors at baseline and after 3, 6, 24, 72 hours, 7 and 14 days at room temperature (RT) and 4 ºC, and after 14 days, 1, 3 and 6 months at – 20 ºC and – 80 ºC.

**Results:**

At RT, changes appeared at 6 hours for TEA and H_2_O_2_; 24 hours for Lip, CK, ADA and CUPRAC; and 72 hours for LD, Lact, FRAS, UA and AOPP. At 4 ºC changes were observed after 6 hours for TEA and H_2_O_2_; 24 hours for Lip and CUPRAC; 72 hours for CK; and 7 days for LD, FRAS and UA. At – 20 ºC changes appeared after 14 days for AST, Lip, CK and LD; and 3 months for TEA and H_2_O_2_. At – 80 ºC observed changes were after 3 months for TEA and H_2_O_2_.

**Conclusions:**

In short-term storage, the analytes were more stable at 4 ºC than at room temperature, whereas in long-term storage they were more stable at - 80 ºC than at – 20 ºC.

## Introduction

Interest in saliva assays for clinical purposes has increased during recent years because this fluid has important advantages: its collection is easy, does not produce evident stress or pain and does not require expensive material or medical personnel. The main areas in which saliva can be used for testing are psychology and stress research, endocrinology, occupational and sports medicine, drug monitoring, metabolism and oxidative status evaluation, immunology and inflammation (*1*).

The use of salivary biomarkers for diagnostic purposes would be of benefit if standardised procedures for saliva collection were used, as well as the validation/verification of the methods performed in saliva. In addition, knowledge of how the storage conditions can affect the stability of measured analytes is of major importance, especially as saliva is usually less stable than plasma (due to bacterial multiplication, viscosity or extensive proteolytic cleavage by endogenous and exogenous proteases) (*2*). Stability would be of particular importance when retrospective studies or studies involving multiple experimental sampling time-points are designed, since they usually involve the storage of samples, and an inappropriate temperature can affect enzymatic activities in saliva samples during sampling and storage (*3*).

Salivary α-amylase (AMY) increases in situations of acute stress and activation of the sympathetic nervous system (SNS), due to different psychological causes or physical efforts (*4*). Changes in salivary cholinesterase (CHE) activity have been described in Alzheimer’s disease and they have been related to situations of stress, although it is traditionally measured as a biomarker of anti-CHE insecticides exposure (*5*, *6*). Salivary lipase (Lip) secretion also seems to be promoted by the activation of the SNS (*7*). The total esterase activity (TEA) of saliva comprises several enzymatic activities, and it is increased in situations of physical stress (*8*). Creatine kinase (CK), aspartate transaminase (AST) and lactate dehydrogenase (LD) can increase in human saliva in conditions such as intensive exercise (*9*). Lactate (Lact) is considered a marker of muscle function and its quantification in saliva is important in internal and sport medicine to monitor the maximum performance level of athletes (*10*). Adenosine deaminase (ADA) is involved in various processes related with the immune system, it is considered a biomarker of inflammation and it has been found to change in saliva in oral diseases (*11*).

In situations of oxidative stress, reactive oxygen species (ROS) are produced in high amounts that cannot be removed by antioxidants. Total antioxidant capacity (TAC) represents the antioxidant status of a sample and comprises the sum of the concentrations of almost all non-enzymatic antioxidants (*12*). It can be evaluated in saliva by different assays, such as ferric reducing ability of saliva (FRAS), cupric reducing antioxidant capacity (CUPRAC), and the 6-hydroxy-2,5,7,8-tetramethylchroman-2-carboxylic acid (Trolox) equivalent antioxidant capacity (TEAC) (*13*). The Trolox equivalent antioxidant capacity, FRAS and CUPRAC are assays based on the ability of antioxidants present in a sample to reduce or inhibit oxidized products generated in the assay (*14*). The Trolox equivalent antioxidant capacity measures the capacity of the sample to reduce the 2,2’-azino-bis(3-ethylbenz-thiazoline-6-sulfonic acid) (ABTS) radical, whereas FRAS and CUPRAC measure the ability of a sample to reduce Fe^3+^ and Cu^2+^ to Fe^2+^ and Cu^1+^, respectively. They have been evaluated in saliva in patients with diseases such as oral lichen planus (*13*). In addition, individual components of the antioxidant system such as uric acid (UA) and catalase (CAT) can be measured. Uric acid is the final metabolite of purines and represents approximately 70% of salivary TAC (*15*). It can increase in hypoxia due to the appearance of oxidant metabolites and there is evidence that salivary UA is altered in diseases such as oral lichen planus or diabetes (*13*, *16*). Catalase is an enzyme capable of removing ROS from saliva and its activity is altered in patients with different diseases such as human immunodeficiency virus (*17*). Components of the oxidant system can also be measured in saliva, namely the advanced oxidation protein products (AOPP) and hydrogen peroxide (H_2_O_2_). Advanced oxidation protein products represent a sensitive biomarker of oxidative-modified proteins and it has been measured in human saliva before and after acute resistance exercise (*18*). Hydrogen peroxide is a reactive species produced during normal metabolism, with increased concentrations found in situations of inflammation and tissue damage (*19*).

Studies on the stability for some of these analytes already have been published; such as stability of AMY at room temperature (RT), 4 ºC and after freeze-thaw cycle; stability of LD and AST at RT, 4 ºC and – 20 °C for 3 months and at – 80 ºC for 28 days, or the stability of AOPP for 4 weeks at – 20 ºC and – 80 ºC (*2*, *3*, *20*-*25*). However, long-term studies longer than 3 months in which different storage conditions were compared have not been published. In addition, to the authors’ knowledge, there have not been studies about the stability of CHE, Lip, TEA, CK, Lact, ADA, TEAC, UA, CAT and H_2_O_2_ in human saliva under different conditions of storage.

Our hypothesis was that the analytes in saliva can have different changes depending of the sample storage conditions and used times. In this report, we aimed to examine the stability of various analytes (AMY, CHE, Lip, TEA, CK, AST, LD, Lact, ADA, TEAC, FRAS, CUPRAC, UA, CAT, AOPP and H_2_O_2_) in saliva under different storage conditions.

## Material and methods

### Subjects

This experimental study was conducted at the Interdisciplinary Laboratory of Clinical Analyses of the University of Murcia, Spain, from June to December 2017. The experimental protocol was approved by the Investigation Ethics Committee of the University of Murcia (Spain) and followed the Declaration of Helsinki of the World Medical Association for research with humans.

Twelve subjects, six men (29-58 years) and six women (28-56 years) participated in this study. They were healthy volunteers from the personnel of Murcia University. All participants filled a questionnaire in which they were asked about habits, the presence and description of any acute or chronic disease, any type of symptom in the days before the experiment took place, or whether they were receiving or they had recently received any medical treatment. The inclusion criteria for the participants were that they should be adults without any acute or chronic disease, not under physician’s care for any disease for the last 6 months, not receiving any medication (including hormones, steroids or non-steroidal anti-inflammatories), food supplements (vitamins or sport supplements) and not being smokers or alcohol consumers. In addition, subjects should not have oral diseases, such as gingivitis or periodontitis, after complete examination of the oral cavity performed by an oral medicine professional.

### Methods

The participants themselves performed sample collection. All participants received detailed information by oral communication and written guidelines about the aims and experimental protocol, the saliva collection procedure, and signed written consent. They were informed about the need to avoid coughing or clearing the throat into the collection tube and were to abstain from brushing teeth or using mouthwash, ingesting any food or chewing gum for 1 hour before saliva collection.

The participants rinsed their mouth with water five minutes before saliva collection. Then, unstimulated saliva was collected by passive drool in the absence of chewing movements into 10 mL plain tubes (Proquilab, Murcia, Spain). Collection started in all subjects at 9:30 am and lasted between 2 and 5 minutes. The volunteers sat in a relaxed position throughout the sampling procedure to avoid any stress. Between 3 to 5 mL of saliva was obtained from each participant, all samples were checked for blood contamination by visual inspection and no reddish samples indicating blood contamination were included in the study. Immediately after collection, the saliva samples were centrifuged (Universal 320R, Hettich, Tuttlingen, Germany) at 5000xg and 4 ºC for 5 minutes, then the supernatant was collected and divided into aliquots, discarding the sediment. To evaluate the effects of different storage conditions, 19 aliquots of each sample were prepared: (a) 1 aliquot was freshly analysed (baseline) and used as the reference value for all analytes; (b) 4 aliquots of each sample were stored at room temperature (RT); (c) 6 aliquots were refrigerated at 4 ºC, (d) 4 were frozen at – 20 ºC and (e) 4 were frozen at – 80 ºC. The aliquots stored at RT were analysed at 3 (T1), 6 (T2), 24 (T3) and 72 (T4) hours after sampling; the refrigerated aliquots were analysed at T1, T2, T3 and T4, and in addition 7 (T5) and 14 days (T6) from collection. Finally, the aliquots stored at – 20 ºC and at – 80 ºC were analysed 14 days (T6), 1 (T7), 3 (T8) and 6 months (T9) from collection.

The analytical methods used, which were adapted in the authors’ laboratory for saliva samples, as well as their fundamentals, details of the reagents and analytical performance, appear in Table 1. All assays were performed on an automated biochemistry analyser (Olympus AU400, Olympus Diagnostica GmbH, Ennis, Ireland) at 37 ºC. Manufacturers’ control solutions of two different values were used for the quality control analysis of AMY, Lip, CK, AST, LD, Lact and UA (Beckman Coulter, lot 0037 and 0038) and one control solution was used for ADA (Diazyme, DZ177A-Con). Daily in-house controls of two different values were used for analysis of FRAS, TEAC, CUPRAC, CAT, AOPP and H_2_O_2_. The control for FRAS was a ferric chloride hexahydrate solution, for TEAC and CUPRAC a Trolox solution, an enzyme solution for CAT, Chloramine-T solution for AOPP, and a H_2_O_2_ solution for H_2_O_2_ assay. A single measurement was made in all cases since all analytical methods showed an intra-assay imprecision lower than 15%, which indicated adequate assay repeatability.

**Table 1:** Analytical methods used and results of their analytical performance in human saliva

**Method**	**Reference in which the method was validated for saliva or basis of the method (in those assays in which validation was performed specifically for this research)**	**Manufacturer or reagents (in home-made assays)**		**Intra-assay** **CV (%)**	**Inter-assay** **CV (%)**	**Linearity (R^2^)**	**LLOD**
		**R1**	**R2**				
α-amylase	(*24*)	Beckman Coulter^a^		< 3.0	< 3.0	> 0.99	11.7 U/L
Cholinesterase	Hydrolysis of ATCI to thiocholine in presence DTNB; non-enzymatic hydrolysis of ATCI must be subtracted	1mM DTNB, in 0.1M phosphate buffer pH 7.5	55mM ATCI	4.5^b^	6.2^b^	0.90^c^	1.4^d^ µM/mL/min
Lipase	1,2-Diglyceride is hydrolyzed to 2-monoglyceride and fatty acid. The 2- monoglyceride is then measured by coupled enzyme reactions catalyzed by monoglyceride lipase, glycerol kinase, glycerol phosphate oxidase and peroxidase	Beckman Coulter^a^		4.3^b^	5.7^b^	> 0.99^c^	1.0^d^ U/L
Total esterase activity	(*8*)	0.1M Tris-HCl buffer pH 8.0	4.55mM 4-nitrophenyl acetate	2.46	5.18	> 0.99	0.8 U/L
Creatin kinase	(*25*)	Beckman Coulter^a^		< 8.0	< 9.0	> 0.99	2.0 U/L
Aspartate transaminase	(*25*)	Beckman Coulter^a^		< 6.0	< 11.0	> 0.99	3.0 U/L
Lactate dehydrogenase	(*25*)	BioSystems^e^		< 1.0	< 3.0	> 0.99	10.0 U/L
Adenosine deaminase	Deamination of adenosine to inosine, which is converted to hypoxanthine by purine nucleoside phosphorylase (PNP). Hypoxanthine is then converted to uric acid and hydrogen peroxide (H_2_O_2_) by xanthine oxidase (XOD). H_2_O_2_ is further reacted with N-Ethyl-N-(2-hydroxy-3-sulfopropyl)-3-methylaniline (EHSPT) and 4-aminoantipyrine (4-AA) in the presence of peroxidase (POD) to generate quinone dye	Diazyme^f^		7.3^b^	6.1^b^	> 0.99^c^	0.07^d^ U/L
Lactate	(*10*)	Beckman Coulter^a^		2.86	5.23	0.99	0.01 mM
**Method**	**Reference in which the method was validated for saliva or basis of the method (in those assays in which validation was performed specifically for this research)**	**Manufacturer or reagents (in home-made assays)**		**Intra-assay** **CV (%)**	**Inter-assay** **CV (%)**	**Linearity (R^2^)**	**LLOD**
		**R1**	**R2**				
FRAS	Reduction of Fe^3+^ to Fe^2+^ by the antioxidants present in the sample	0.7mM of TPTZ, and 1.5mM of FeCl_3_·6H_2_O in acetate buffer	None	0.89^b^	2.25 ^b^	0.99^c^	0.031 mM
TEAC	Reduction of ABTS radical to ABTS by the antioxidants present in the sample	2mM of ABTS, 0.25μM of HRP, and 40μM of H_2_O_2_ in 50mM of phosphate buffer	None	1.91^b^	4.51^b^	0.99^c^	0.09 mM
CUPRAC	Reduction of Cu^2+^ to Cu^1+^ by the antioxidants present in the sample	0.25mM of BCS in 10mM of phosphate buffer	0.5mM of CuSO_4_ in ultrapure water	0.60^b^	1.25^b^	0.99^c^	0.003 mM
Uric acid	(*10*)	Beckman Coulter^a^		0.57	6.41	>0.99	0.22 µmol/L
Catalase	Inhibition of colour development in a H_2_O_2_-producing urate assay	2mM of DHBS, 0.5mM of AP, 50mM of Fe(CN)_6_·3H_2_O, 28 U/L of uricase, and 200 U/L of HRP in 50mM of phosphate buffer	500mM of uric acid	2.09^b^	13.03^b^	0.91^c^	0.03 units/mL
AOPP	Producing solutions which in the presence of potassium iodide absorb at 340nm	0.059 M of KI in ultrapure water	Acetic acid, 50% (v/v)	1.48^b^	6.25^b^	0.98^c^	3.67 μM
H_2_O_2_	Production of diamine (yellow-coloured oxidation product) which absorbs at 450 nm	0.7mM of TMB and 25mU of HRP in 100mM of phosphate buffer	500mM of sulphuric acid	3.43^b^	16.58^b^	0.95^c^	0.01 μM
CV - coefficient of variation. R^2^ - coefficient of determination. LLOD - lower limit of detection. R1 - reagent 1. R2 - reagent 2. ATCI - acetylthiocholine iodide. DTNB - 5,5’-dithiobis-2-nitrobenzoic acid. TPTZ – tripyridyltriazine. FeCl_3_·6H_2_O - ferric chloride hexahydrate. FeSO_4_·7H_2_O - ferrous sulfate heptahydrate. ABTS - 2,2’-azino-bis-3-ethylbenzthiazoline-6-sulfonic acid. HRP - horseradish peroxidase. BCS - bathocuproinedisulfonic acid disodium salt. CuSO_4_ – copper (II) sulphate. DHBS - 3,5-dichloro-2-hydroxybenzenesulphonate. AP - 4-aminophenazone. K_4_Fe(CN)_6_·3H_2_O - potassium hexacyanoferrate II. KI - potassium iodide. TMB, - 3,5,3050-tetramethylbenzidine. ^a^Beckman Coulter Inc, Fullerton, CA, USA. ^b^Mean of CVs for 6 repeated measurements of three pooled saliva samples of different concentrations as follows: standard deviation from the 6 measurements divided by mean and multiplied by 100. ^c^Mean of R^2^ measured after linearity under dilution of two saliva samples. ^d^Calculated as mean + 2 standard deviations of 20 replicates of the zero standard. ^e^BioSystems, Barcelona, Spain. ^f^Diazyme Laboratories, Poway, CA, USA.							

### Statistical analysis

Descriptive statistical procedures and spreadsheets (Excel 2000, Microsoft Corporation, Redmond, USA) and software (Graph Pad Prism, Version 5 for Windows, Graph Pad Software Inc, San Diego, USA, and IBM SPSS statistic for Windows, version 24.0, IBM Corp., Armonk, USA) were used. Since only 12 data were included, normality was not assumed. Therefore, the values of the analytes at different times and conditions after storage were compared with aliquots analysed immediately using the Friedman test, followed by Dunn’s multiple comparison test. The results were considered significant when P < 0.05.

## Results

The results for the stability of the studied analytes, under different storage conditions, are shown in Table 2.

**Table 2:** Stability results for 12 different saliva analytes obtained after measuring samples at different processing time and under different storage conditions

**Analyte** **(unit)**	**T0**	**Temperature**		**T1**	**T2**	**T3**	**T4**	**T5**	**T6**	**T7**	**T8**	**T9**
				58,570 (47,055 – 101,200)	71,340 (48,460 – 94,915)	74,920 (44,770 – 92,445)	79,850 (48,175 -100,690)	-	-	-	-	-
		**RT**	**Variation (%)**	1.9	24.1	30.4	38.9	-	-	-	-	-
			**P**	> 0.999	> 0.999	> 0.999	> 0.999	-	-	-	-	-
				78,810 (48,060 – 102,305)	68,670 (41,470 – 90,655)	76,480 (39,935 – 95,885)	79,970 (47,300 – 101,295)	76,540 (47,395 – 99,160)	81,000 (48,325 – 100,705)	-	-	-
		**4 ºC**	**Variation (%)**	37.1	19.5	33.1	39.2	33.2	40.9	-	-	-
			**P**	0.494	> 0.999	> 0.999	0.769	> 0.999	> 0.999	-	-	-
**AMY (U/L)**	57,470 (39,615 – 101,310)			-	-	-	-	-	79,870 (47,165 – 100,730)	68,840 (40,750 – 89,215)	74,160 (42,280 – 97,095)	77,610 (47,965 – 99,245)
		**- 20 ºC**	**Variation (%)**	-	-	-	-	-	39.0	19.8	29.0	35.0
			**P**	-	-	-	-	-	> 0.999	0.054	> 0.999	> 0.999
				-	-	-	-	-	79,300 (47,730 – 100,625)	68,140 (39,715 – 87,200)	71,250 (42,175 – 89,315)	77,460 (49,580 – 104,120)
		**- 80 ºC**	**Variation (%)**	-	-	-	-	-	38.0	18.6	24.0	34.8
			**P**	-	-	-	-	-	> 0.999	0.156	0.248	> 0.999
				7.4 (3.7 -10.3)	5.5 (0.1 – 7.7	5.3 (2.6 - 11.1)	5.3 (3.6 – 11.9)	-	-	-	-	-
		**RT**	**Variation (%)**	2.8	23.6	26.4	26.4	-	-	-	-	-
			**P**	> 0.999	> 0.999	> 0.999	> 0.999	-	-	-	-	-
				7.2 (3.6 -10.3)	6.6 (3.2 – 8.8)	11.2 (7.8 – 14.5)	7.1 (4.0 – 10.7)	11.4 (9.3 – 14.7)	7.4 (6-6 – 10.3)	-	-	-
		**4 ºC**	**Variation (%)**	0.0	8.3	55.6	1.4	58.3	2.8	-	-	-
			**P**	> 0.999	> 0.999	0.223	> 0.999	0.097	> 0.999	-	-	-
**CHE (µM/mL/min)**	7.2 (2.6 - 9.5)			-	-	-	-	-	4.9 (3.1 – 8.3)	6.7 (4.2 – 8.0)	5.9 (4.2 – 7.2)	4.0 (0.6 – 4.8)
		**- 20 ºC**	**Variation (%)**	-	-	-	-	-	31.9	6.9	18.1	44.4
**Analyte** **(unit)**	**T0**	**Temperature**		**T1**	**T2**	**T3**	**T4**	**T5**	**T6**	**T7**	**T8**	**T9**
			**P**	-	-	-	-	-	> 0.999	> 0.999	> 0.999	> 0.999
				-	-	-	-	-	7.2 (6.1 – 11.3)	8.1 (5.8 – 11.0)	6.9 (4.9 – 10.1)	4.5 (3.8 – 7.3)
		**- 80 ºC**	**Variation (%)**	-	-	-	-	-	0.0	12.5	4.2	37.5
			**P**	-	-	-	-	-	> 0.999	> 0.999	> 0.999	> 0.999
				10.6 (4.5 – 25.5)	11.6 (4.3 – 15.9)	4.4 (3.6 – 8.2)	4.1 (3.6 -4.7)	-	-	-	-	-
		**RT**	**Variation (%)**	20.9	13.4	67.2	69.4	-	-	-	-	-
			**P**	> 0.999	> 0.999	< 0.001	< 0.001	-	-	-	-	-
				14.3 (5.1 -23.3)	15.7 (3.7 - 22.1)	6.3 (2.4 – 16)	2.1 (0.4 - 3.3)	1.7 (1.4 - 4.1)	0.6 (0.5 - 3.9)	-	-	-
		**4 ºC**	**Variation (%)**	6.7	17.2	53.0	84.3	87.3	95.5	-	-	-
			**P**	> 0.999	> 0.999	< 0.001	< 0.001	<0.001	< 0.001	-	-	-
**Lip (U/L)**	13.4 (7.3 - 33.2)			-	-	-	-	-	3.8 (0.9 – 8.0)	2.0 (1.4 – 14.0)	2.0 (1.2 - 3.5)	3.5 (2.4 - 10.6)
		**- 20 ºC**	**Variation (%)**	-	-	-	-	-	71.6	85.1	85.1	73.9
			**P**	-	-	-	-	-	0.018	0.017	< 0.001	0.031
				-	-	-	-	-	12.5 (3.5 - 32.4)	9.9 (2.7 - 36.4)	11.6 (4.1 - 34.7)	12.9 (4.5 - 54.4)
		**- 80 ºC**	**Variation (%)**	-	-	-	-	-	6.7	26.1	13.4	3.7
			**P**	-	-	-	-	-	> 0.999	> 0.999	> 0.999	> 0.999
				20.1 (14.4 -48.8)	15.7 (12.4 - 39.1)	27.7 (23.8 - 32.4)	18.1 (16.4 - 25.4)	-	-	-	-	-
		**RT**	**Variation (%)**	9.9	29.3	24.5	18.6	-	-	-	-	-
			**P**	> 0.999	0.002	> 0.999	0.035	-	-	-	-	-
				19.7 (15.7 - 51.3)	16.4 (13.3 - 42.4)	28.8 (25.4 - 57.4)	14.8 (12.2 - 27.2)	26.5 (20.4 -42.6)	23.2 (18.4 - 34.2)	-	-	-
		**4 ºC**	**Variation (%)**	11.5	26.5	29.2	33.7	18.9	4.3	-	-	-
			**P**	> 0.999	0.041	> 0.999	< 0.001	> 0.999	> 0.999	-	-	-
**Analyte** **(unit)**	**T0**	**Temperature**		**T1**	**T2**	**T3**	**T4**	**T5**	**T6**	**T7**	**T8**	**T9**
**TEA (U/L)**	22.3 (15.6 - 59.4)			-	-	-	-	-	19.9 (15.6 - 49.7)	24.4 (17 - 56.9)	16.8 (9.9 -35.4)	21.5 (13.9 -43.4)
		**- 20 ºC**	**Variation (%)**	-	-	-	-	-	10.3	9.7	24.5	3.4
			**P**	-	-	-	-	-	> 0.999	> 0.999	< 0.001	0.315
				-	-	-	-	-	20.5 (15.4 - 50.9)	24.3 (17.8 - 56.7)	13.6 (9.0 -34.2)	21.0 (15.6 -53)
		**- 80 ºC**	**Variation (%)**	-	-	-	-	-	8.1	9.0	38.9	5.6
			**P**	-	-	-	-	-	> 0.999	> 0.999	< 0.001	> 0.999
				9.1 (3.6 - 21.6)	10.0 (3.8 - 20.4)	5.5 (1.5 - 9.8)	6.0 (2.4 - 11.2)	-	-	-	-	-
		**RT**	**Variation (%)**	45.5	40.1	67.2	63.9	-	-	-	-	-
			**P**	> 0.999	0.248	0.002	0.003	-	-	-	-	-
				11.7 (5.8 - 21.4)	11.5 (6.6 - 16.5)	10.9 (4.7 - 18.0)	5.7 (2.7 - 10.1)	4.4 (0.9 - 7.8)	2.0 ((- 1.0) -7.9)	-	-	-
		**4 ºC**	**Variation (%)**	29.8	30.7	34.6	66.0	70.8	88.0	-	-	-
			**P**	> 0.999	> 0.999	> 0.999	0.006	<0.001	< 0.001	-	-	-
**CK (U/L)**	16.6 (7.00 - 33.2)			-	-	-	-	-	7.0 (3.4 - 10.8)	5.9 (4.7 -10.2)	7.2 (5.7 - 11.5)	6.9 (4.0 - 8.5)
		**- 20 ºC**	**Variation (%)**	-	-	-	-	-	57.8	64.8	56.6	58.4
			**P**	-	-	-	-	-	0.003	0.043	0.045	0.020
				-	-	-	-	-	12.9 (6.7 - 22.4)	13.6 (8.7 - 20.7)	15.9 (8.1 - 20.5)	13.6 (10.1 - 20.1)
		**- 80 ºC**	**Variation (%)**	-	-	-	-	-	22.6	18.1	4.2	18.4
			**P**	-	-	-	-	-	> 0.999	> 0.999	> 0.999	> 0.999
				11.2 (9.0 - 25.5)	11.5 (10.2 - 24.0)	15.3 (12.4 - 24.7)	22.2 (15.3 - 32.5)	-	-	-	-	-
		**RT**	**Variation (%)**	7.1	4.6	27.0	83.8	-	-	-	-	-
**Analyte** **(unit)**	**T0**	**Temperature**		**T1**	**T2**	**T3**	**T4**	**T5**	**T6**	**T7**	**T8**	**T9**
			**P**	> 0.999	> 0.999	> 0.999	> 0.999	-	-	-	-	-
				11.7 (9.5 - 25.2)	12.7 (10.5 - 24.4)	12.8 (10.3 - 23.6)	10.3 (8.3 - 24.1)	10.7 (8.4 - 29.3)	10.3 (8.2 - 24.7)	-	-	-
		**4 ºC**	**Variation (%)**	3.3	5.0	6.2	14.5	11.2	14.9	-	-	-
			**P**	> 0.999	> 0.999	> 0.999	0.760	0.508	0.222	-	-	-
**AST (U/L)**	12.1 (10.1 - 26.7)			-	-	-	-	-	9.7 (7.7 - 17.2)	9.1 (5.9 - 11.3)	7.9 (6.1 - 11.5)	5.2 (2.8 - 6.0)
		**- 20 ºC**	**Variation (%)**	-	-	-	-	-	19.5	24.9	34.4	57.3
			**P**	-	-	-	-	-	0.028	< 0.001	< 0.001	< 0.001
				-	-	-	-	-	12.8 (11.0 - 25.1)	13.1 (11.1 - 26.1)	13.5 (11.4 - 26.1)	13.9 (9.7 - 25.0)
		**- 80 ºC**	**Variation (%)**	-	-	-	-	-	6.2	8.3	12.0	15.4
			**P**	-	-	-	-	-	> 0.999	> 0.999	> 0.999	0.666
				338 (214 -409)	339 (212 – 399)	276 (189 – 344)	204 (139 – 295)	-	-	-	-	-
		**RT**	**Variation (%)**	0.3	0.6	18.1	39.3	-	-	-	-	-
			**P**	> 0.999	> 0.999	0.365	0.006	-	-	-	-	-
				333 (224- 402)	336 (193 – 404)	320 (211 – 383)	220 (172 – 338)	196 (156 – 302)	131 (77 – 261)	-	-	-
		**4 ºC**	**Variation (%)**	1.0	0.1	5.0	34.8	41.7	61.1	-	-	-
			**P**	> 0.999	> 0.999	> 0.999	0.222	0.006	< 0.001	-	-	-
**LD (U/L)**	337 (221-403)			-	-	-	-	-	69 (27 – 168)	54 (18 – 97)	27 (5 – 59)	31 (15 – 59)
		**- 20 ºC**	**Variation (%)**	-	-	-	-	-	79.5	84.0	92.1	90.9
			**P**	-	-	-	-	-	< 0.001	< 0.001	< 0.001	< 0.001
				-	-	-	-	-	323 (214 – 391)	321 (223 – 399)	335 (234 – 414)	370 (279 – 445)
**Analyte** **(unit)**	**T0**	**Temperature**		**T1**	**T2**	**T3**	**T4**	**T5**	**T6**	**T7**	**T8**	**T9**
		**- 80 ºC**	**Variation (%)**	-	-	-	-	-	4.0	1.8	0.6	10.0
			**P**	-	-	-	-	-	> 0.999	> 0.999	> 0.999	> 0.999
				0.5 (0.4 - 1.3)	0.5 (0.4 - 1.3)	0.3 (0.1 - 1.2)	0.01((- 0.003) -0.04)	-	-	-	-	-
		**RT**	**Variation (%)**	1.0	1.0	58.2	98.1	-	-	-	-	-
			**P**	> 0.999	> 0.999	0.634	0.003	-	-	-	-	-
				0.5 (0.4 - 1.2)	0.5 (0.4 - 1.2)	0.4 (0.3 -1.3)	0.4 (0.3 - 1.4)	0.5 (0.4 -1.5)	0.5 (0.1 - 0.7)	-	-	-
		**4 ºC**	**Variation (%)**	1.9	1.0	15.5	17.5	8.7	9.7	-	-	-
			**P**	> 0.999	> 0.999	> 0.999	> 0.999	>0.999	> 0.999	-	-	-
**Lact** **(mmol/L)**	0.5 (0.4 - 1.2)			-	-	-	-	-	0.5 (0.4 - 1.2)	0.5 (0.4 - 1.1)	0.5 (0.4 - 1.2)	0.5 (0.4 - 1.2)
		**- 20 ºC**	**Variation (%)**	-	-	-	-	-	1.0	10.7	5.8	3.9
			**P**	-	-	-	-	-	> 0.999	> 0.999	> 0.999	> 0.999
				-	-	-	-	-	0.5 (0.4 - 1.2)	0.5 (0.4 - 1.2)	0.5 (0.4 - 1.2)	0.5 (0.4 -1.2)
		**- 80 ºC**	**Variation (%)**	-	-	-	-	-	1.0	6.8	1.9	3.9
			**P**	-	-	-	-	-	> 0.999	> 0.999	> 0.999	> 0.999
				1.2 (0.8 - 1.7)	1.3 (1.0 - 1.9)	2.0 (1.2 -2.2)	1.1 (0.9 - 1.8)	-	-	-	-	-
		**RT**	**Variation (%)**	18.2	35.4	101	6.1	-	-	-	-	-
			**P**	> 0.999	0.059	0.007	> 0.999	-	-	-	-	-
				1.1 (0.9 - 1.6)	1.2 (0.8 - 1.8)	1.0 (0.8 - 1.9)	0.9 (0.6 - 1.7)	0.8 (0.6 -1.2)	0.6 (0.5 - 1.2)	-	-	-
		**4 ºC**	**Variation (%)**	12.1	21.2	4.0	6.1	20.2	37.4	-	-	-
			**P**	> 0.999	0.734	> 0.999	> 0.999	>0.999	> 0.999	-	-	-
**Analyte** **(unit)**	**T0**	**Temperature**		**T1**	**T2**	**T3**	**T4**	**T5**	**T6**	**T7**	**T8**	**T9**
**ADA** **(U/L)**	1.0 (0.7 - 1.6)			-	-	-	-	-	1.0 (0.5 - 1.7)	0.8 (0.3 - 1.3)	0.5 (0.2 - 1.1)	0.6 (0.2 - 0.8)
		**- 20 ºC**	**Variation (%)**	-	-	-	-	-	2.0	15.2	45.5	41.4
			**P**	-	-	-	-	-	> 0.999	> 0.999	0.189	0.064
				-	-	-	-	-	1.2 (0.8 - 1.8)	1.2 (0.8 - 1.8)	1.0 (0.7 - 1.5)	1.2 (1.0 - 2.0)
		**- 80 ºC**	**Variation (%)**	-	-	-	-	-	17.2	16.2	2.0	18.2
			**P**	-	-	-	-	-	> 0.999	> 0.999	> 0.999	0.291
				0.2 (0.2 - 0.3)	0.2 (0.2 - 0.3)	0.1 (0.1 - 0.2)	0.2 (0.1 - 0.2)	-	-	-	-	-
		**RT**	**Variation (%)**	31.6	26.2	13.9	9.1	-	-	-	-	-
			**P**	0.701	0.529	0.582	0.582	-	-	-	-	-
				0.2 (0.2 - 0.3)	0.2 (0.2 - 0.3)	0.2 (0.1 - 0.2)	0.2 (0.1 - 0.2)	0.2 (0.2 -0.3)	0.2 (0.1 - 0.2)	-	-	-
		**4 ºC**	**Variation (%)**	29.5	24.1	4.6	7.4	17.1	10.5	-	-	-
			**P**	> 0.999	> 0.999	> 0.999	> 0.999	0.064	> 0.999	-	-	-
**TEAC** **(mM)**	0.2 (0.1 – 0.2)			-	-	-	-	-	-	0.2 (0.1 - 0.2)	0.2 (0.1 - 0.2)	0.2 (0.1 - 0.3)
		**- 20 ºC**	**Variation (%)**	-	-	-	-	-	-	6.0	6.8	33.8
			**P**	-	-	-	-	-	-	> 0.999	> 0.999	0.457
				-	-	-	-	-	-	0.2 (0.1 - 0.2)	0.2 (0.1 - 0.3)	0.2 (0.2 - 0.3)
		**- 80 ºC**	**Variation (%)**	-	-	-	-	-	-	8.3	11.0	32.0
			**P**	-	-	-	-	-	-	> 0.999	0.291	0.841
				0.4 (0.3 - 0.5)	0.4 (0.3 - 0.5)	0.4 (0.3 - 0.5)	0.3 (0.2 - 0.3)	-	-	-	-	-
		**RT**	**Variation (%)**	3.3	9.1	9.5	13.3	-	-	-	-	-
**Analyte** **(unit)**	**T0**	**Temperature**		**T1**	**T2**	**T3**	**T4**	**T5**	**T6**	**T7**	**T8**	**T9**
			**P**	> 0.999	0.669	> 0.999	< 0.001	-	-	-	-	-
				0.4 (0.2 - 0.5)	0.3 (0.2 - 0.5)	0.4 (0.3 - 0.5)	0.3 (0.3 - 0.4)	0.3 (0.2 -0.4)	0.4 (0.3 - 0.4)	-	-	-
		**4 ºC**	**Variation (%)**	3.0	1.3	2.0	4.5	9.4	4.3	-	-	-
			**P**	> 0.999	> 0.999	> 0.999	0.555	0.018	0.047	-	-	-
**FRAS** **(mM)**	0.3 (0.3 - 0.5)			-	-	-	-	-	0.3 (0.3 - 0.5)	0.4 (0.3 - 0.5)	0.3 (0.3 - 0.5)	0.3 (0.3 - 0.5)
		**- 20 ºC**	**Variation (%)**	-	-	-	-	-	0.1	10.4	4.8	0.3
			**P**	-	-	-	-	-	> 0.999	> 0.999	> 0.999	> 0.999
				-	-	-	-	-	0.3 (0.2 - 0.5)	0.3 (0.2 - 0.5)	0.3 (0.2 - 0.5)	0.3 (0.2 - 0.5)
		**- 80 ºC**	**Variation (%)**	-	-	-	-	-	0.1	0.6	1.3	2.1
			**P**	-	-	-	-	-	> 0.999	> 0.999	> 0.999	0.394
				0.2 (0.2 - 0.2)	0.2 (0.1 - 0.2)	0.2 (0.1 - 0.2)	0.2 (0.1 - 0.2)	-	-	-	-	-
		**RT**	**Variation (%)**	1.1	1.5	0.1	27.2	-	-	-	-	-
			**P**	> 0.999	> 0.999	0.010	< 0.001	-	-	-	-	-
				0.2 (0.2 - 0.2)	0.2 (0.1 - 0.2)	0.2 (0.1 - 0.2)	0.2 (0.1 - 0.2)	0.2 (0.1 -0.2)	0.2 (0.1 - 0.2)	-	-	-
		**4 ºC**	**Variation (%)**	0.8	0.1	5.9	12.7	14.3	10.1	-	-	-
			**P**	> 0.999	0.794	0.002	< 0.001	<0.001	< 0.001	-	-	-
**CUPRAC (mM)**	0.2 (0.2 - 0.3)			-	-	-	-	-	0.2 (0.2 - 0.2)	0.2 (0.2 - 0.2)	0.2 (0.2 - 0.2)	0.2 (0.2 - 0.3)
		**- 20 ºC**	**Variation (%)**	-	-	-	-	-	1.2	0.3	3.3	2.1
			**P**	-	-	-	-	-	> 0.999	> 0.999	0.162	> 0.999
				-	-	-	-	-	0.2 (0.2 - 0.2)	0.2 (0.2 - 0.3)	0.2 (0.2 - 0.2)	0.2 (0.2 - 0.3)
**Analyte** **(unit)**	**T0**	**Temperature**		**T1**	**T2**	**T3**	**T4**	**T5**	**T6**	**T7**	**T8**	**T9**
		**- 80 ºC**	**Variation (%)**	-	-	-	-	-	0.1	3.7	0.2	2.4
			**P**	-	-	-	-	-	> 0.999	> 0.999	0.131	> 0.999
				13.1 (9.5 - 16.7)	13.7 (9.5 - 16.7)	11.9 (7.7 - 16.1)	9.5 (5.9 - 12.5)	-	-	-	-	-
		**RT**	**Variation (%)**	2.6	1.3	12.2	28.6	-	-	-	-	-
			**P**	> 0.999	> 0.999	0.084	< 0.001	-	-	-	-	-
				13.7 (9.5 - 17.3)	13.1 (9.5 - 17.3)	13.1 (8.9 - 16.7)	12.5 (8.3 - 16.1)	11.9 7.7 - 14.3)	10.7 (7.1 - 13.7)	-	-	-
		**4 ºC**	**Variation (%)**	1.7	1.5	4.6	10.2	12.1	20.6	-	-	-
			**P**	> 0.999	> 0.999	> 0.999	0.383	0.014	0.002	-	-	-
**Uric acid (µmol/L)**	13.7 (9.5 - 16.1)			-	-	-	-	-	13.7 (9.5 - 18.4)	13.7 (9.5 - 16.7)	14.3 (9.5 - 17.3)	14.3 (10.1 - 17.8)
		**- 20 ºC**	**Variation (%)**	-	-	-	-	-	1.5	2.4	2.8	5.6
			**P**	-	-	-	-	-	> 0.999	> 0.999	> 0.999	0.365
				-	-	-	-	-	13.7 (9.5 - 16.7)	13.1 (9.5 - 16.7)	13.7 (9.5 - 17.3)	14.3 (9.5 - 16.7)
		**- 80 ºC**	**Variation (%)**	-	-	-	-	-	0.2	2.8	0.0	2.8
			**P**	-	-	-	-	-	> 0.999	> 0.999	> 0.999	0.666
				0.2 (0.2 - 0.3)	0.2 (0.2 - 0.3)	0.2 (0.2 - 0.4)	0.4 (0.2 - 0.5)	-	-	-	-	-
		**RT**	**Variation (%)**	2.2	13.3	12.5	50.0	-	-	-	-	-
			**P**	> 0.999	0.960	> 0.999	> 0.999	-	-	-	-	-
				0.2 (0.2 - 0.3)	0.2 (0.2 - 0.3)	0.3 (0.2 - 0.3)	0.2 (0.2 - 0.3)	0.3 (0.2 - 0.4)	0.2 (0.2 - 0.4)	-	-	-
		**4 ºC**	**Variation (%)**	8.9	1.2	2.0	6.5	33.5	0.4	-	-	-
			**P**	> 0.999	> 0.999	> 0.999	> 0.999	>0.999	> 0.999	-	-	-
**Analyte** **(unit)**	**T0**	**Temperature**		**T1**	**T2**	**T3**	**T4**	**T5**	**T6**	**T7**	**T8**	**T9**
**CAT (units/mL)**	0.2 (0.2 – 0.3)			-	-	-	-	-	0.2 (0.2 - 0.3)	0.3 (0.2 - 0.4)	0.3 (0.2 - 0.4)	0.3 (0.2 - 0.4)
		**- 20 ºC**	**Variation (%)**	-	-	-	-	-	8.1	14.1	19.4	26.2
			**P**	-	-	-	-	-	> 0.999	> 0.999	> 0.999	> 0.999
				-	-	-	-	-	0.3 (0.2 - 0.3)	0.3 (0.2 - 0.4)	0.2 (0.2 - 0.4)	0.2 (0.2 - 0.4)
		**- 80 ºC**	**Variation (%)**	-	-	-	-	-	4.0	11.3	2.4	4.8
			**P**	-	-	-	-	-	> 0.999	> 0.999	> 0.999	> 0.999
				103 (80 – 169)	106 (83 – 168)	121 (79 – 147)	59 (41 – 69)	-	-	-	-	-
		**RT**	**Variation (%)**	6.7	3.7	9.8	46.9	-	-	-	-	-
			**P**	> 0.999	> 0.999	> 0.999	0.001	-	-	-	-	-
				116 (81 – 169)	119 (89 – 165)	115 (67 – 142)	102 (60 – 126)	109 (56 – 173)	95 (69 – 123)	-	-	-
		**4 ºC**	**Variation (%)**	4.6	8.1	4.	8.1	1.2	14.2	-	-	-
			**P**	> 0.999	> 0.999	0.914	0.331	>0.999	> 0.999	-	-	-
**AOPP** **(μM)**	111 (76 -180)			-	-	-	-	-	109 (97 – 184)	115 (100 – 186)	102 (81 – 155)	96 (82 – 146)
		**- 20 ºC**	**Variation (%)**	-	-	-	-	-	1.9	4.1	7.3	13.3
			**P**	-	-	-	-	-	> 0.999	> 0.999	> 0.999	> 0.999
				-	-	-	-	-	114 (90 – 185)	116 (98 – 182)	110 (84 – 171)	124 (83 – 207)
		**- 80 ºC**	**Variation (%)**	-	-	-	-	-	2.9	4.7	0.5	11.8
			**P**	-	-	-	-	-	> 0.999	> 0.999	> 0.999	> 0.999
				5.4 (2.1 - 6.6)	6.8 (3.2 -10.6)	5.6 (4.3 -10.8)	4.4 (2.2 - 7.2)	-	-	-	-	-
		**RT**	**Variation (%)**	120.4	178.8	127.3	80.8	-	-	-	-	-
**Analyte** **(unit)**	**T0**	**Temperature**		**T1**	**T2**	**T3**	**T4**	**T5**	**T6**	**T7**	**T8**	**T9**
			**P**	> 0.999	0.011	0.001	> 0.999	-	-	-	-	-
				5.3 (2.4 - 6.4)	7.3 (3.1 -10.2)	8.6 (5.7 -12.2)	6.1 (3.2 - 7.6)	4.2 (3.5 -7.2)	4.5 (2.5 - 8.0)	-	-	-
		**4 ºC**	**Variation (%)**	117.1	198	252.2	146.9	72.2	84.5	-	-	-
			**P**	> 0.999	0.012	< 0.001	0.960	>0.999	> 0.999	-	-	-
**H_2_O_2_** **(μM)**	2.5 (1.7 - 8.9)			-	-	-	-	-	6.8 (2.1 - 9.4)	7.5 (3.1 - 9.0)	9.2 (4.1 -11.8)	7.7 (3.8 -10.8)
		**- 20 ºC**	**Variation (%)**	-	-	-	-	-	176.3	206.9	274.7	212.2
			**P**	-	-	-	-	-	> 0.999	0.248	< 0.001	0.002
				-	-	-	-	-	6.2 (2.5 -10.8)	6.6 (2.8 - 9.0)	7.2 (3.3 -12.4)	9.6 (3.0 -12.2)
		**- 80 ºC**	**Variation (%)**	-	-	-	-	-	153.5	169.8	193.9	291.0
			**P**	-	-	-	-	-	0.804	> 0.999	< 0.001	0.002
Quantitative data is presented as median and interquartile range. T0 - baseline. T1 - 3 hours. T2 - 6 hours. T3 - 24 hours.T4 - 72 hours T5 - 7 days. T6 - 14 days. T7 – 1 month . T8 – 3 months . T9 – 6months. RT - room temperature. AMY - α-amylase. CHE - cholinesterase. Lip - lipase. TEA - total esterase. CK - creatine kinase. AST - aspartate aminotransferase. LD - lactate dehydrogenase. Lact - lactate. ADA - adenosine deaminase. TEAC - Trolox equivalent antioxidant capacity. FRAS - ferric reducing ability. CUPRAC - cupric reducing antioxidant capacity. CAT - catalase. AOPP - advanced oxidation protein products. H_2_O_2_ - hydrogen peroxide. P < 0.05 represents statistical significant difference.												

At RT, AMY, CHE, AST, TEAC and CAT were stable during the whole experimental period (72 hours). The analytes that showed significant decreases were: TEA at 6 hours; Lip, CK and CUPRAC at 24 hours; and LD, Lact, FRAS, UA and AOPP at 72 hours. Significant increases were detected for H_2_O_2_ at 6 hours and for ADA at 24 hours.

At 4 ºC, AMY, CHE, AST, Lact, ADA, TEAC, CAT and AOPP were stable after 14 days of storage. Significant decreases were observed after 6 hours for TEA; after 24 hours for Lip and CUPRAC; after 72 hours for CK; and after 7 days for LD, FRAS and UA. Significant increases were recorded for H_2_O_2_ after 6 hours.

When samples were stored at - 20 ºC, AMY, CHE, Lact, ADA, TEAC, FRAS, CUPRAC, UA, CAT and AOPP were stable for 6 months. Significant decreases were recorded after 14 days of storage for AST, Lip, CK and LD; and after 3 months for TEA. A significant increase was observed for H_2_O_2_ after 3 months of storage.

At - 80 ºC, AMY, CHE, AST, Lip, CK, LD, Lact, ADA, TEAC, FRAS, CUPRAC, UA, CAT and AOPP were stable for 6 months. Significant decreases were observed after 3 months for TEA. A significant increase was detected for H_2_O_2_ at 3 months.

## Discussion

This study found that although there was a variability in the results depending on the studied analyte, in general, in the short-term storage conditions tested, the storage at 4º C provided longer stability for analytes in saliva than at RT. On the other hand, in the long-term storage conditions tested, - 80º C provided longer stability than - 20º C. In the short-term storage conditions, we also included storage for 72 hours at RT and 7 days at 4º C. We are aware that samples are not usually stored in these conditions; however, other researchers in their stability studies have used similar time points and they were included in our study in order to obtain information regarding stability in those conditions (*26*).

Regarding individual analytes, AMY, CHE and ADA were the enzymes that showed fewer changes in the different storage conditions. The high stability of AMY is in accordance with the results of other studies where, for example, AMY was stable for 5 days at RT or for 10 days at RT or 4 ºC (*20*, *21*). Cholinesterase was also stable in all conditions, so it seems that its activity is not affected after storage, although there is a lack of previous reports to compare with. Adenosine deaminase was also stable in most of the conditions with the exception of RT, where it showed a significant increase. Stability of ADA has been studied in porcine saliva and was considered as stable for up to 1 year at - 80 ºC (*27*). The reason why the activity of this enzyme in saliva increases at RT needs to be further elucidated.

Lipase and CK showed a high stability at - 80 ºC; however, in the rest of the storage conditions they showed significant changes. To our knowledge, the stability of Lip and CK in saliva under different storage conditions has not been accessed before, and the reason for lipase and CK enzymatic activity changes in saliva is unknown and should be further studied. Aspartate aminotransferase showed also the highest stability at - 80 ºC, being stable for 6 months. The lack of stability of AST in other storage conditions, such as - 20 ºC, agrees with previous reports (*2*, *3*).

Total esterase and LD were the most labile enzymes in our stability study. It is difficult to know the reason for the changes of TEA, it is likely to have been influenced by the instability of any of the various enzymes that integrate in this total activity (*8*). Lactate dehydrogenase results in our study were similar to other reports that showed a significant decrease of this enzyme in saliva after only 30 min, 3 days and 2 weeks of storage at - 20 ºC (*2*, *3*, *22*). These results could be due to the lability of the LD-4 and LD-5 isoenzymes at - 20 ºC (*28*).

With the exception of H_2_O_2_, the remaining antioxidants biomarkers showed a high stability under freezing conditions. Uric acid has been observed to remain relatively stable during storage, in accordance with previous studies describing stability at - 20 ºC, - 70 ºC and at - 196 ºC in human serum when stored for 1 year (*29*). This can also help to explain the stability observed for the TEAC and FRAS in saliva, both at - 20 ºC and at - 80 ºC, as UA is one of the main contributors to TEAC and FRAS. When oxidant biomarkers were studied, AOPP showed a high level of stability in all conditions, except at RT. Only H_2_O_2_ was very unstable and could only be measured after 3 hours at 4 ºC or 1 month under freezing conditions. Further studies should be made to elucidate the reason for the production of H_2_O_2_ in the saliva samples when they are stored.

This study has some limitations that should be taken into account. Firstly, the study has been made in healthy subjects. It would be of interest in the future to perform further studies also involving subjects with different diseases in order to evaluate the possible differences of stability between samples of healthy and sick subjects. Although in line with previous reports, the number of subjects included in this study can be considered low and ideally a higher number of cases should be included (*21*, *25*, *30*). Therefore, this report should be taken as a pilot study and additional studies would be needed prior to making recommendations about the storage conditions. In addition, in the case of the enzymes, it would have been interesting to study the stability of the different isoenzymes.

It can be concluded that in short-term storage the analytes were more stable at 4 ºC than at room temperature, whereas in long-term storage they were more stable at - 80 ºC than at - 20 ºC.
